# SNX27-driven membrane localisation of OTULIN antagonises linear ubiquitination and NF-κB signalling activation

**DOI:** 10.1186/s13578-021-00659-5

**Published:** 2021-07-27

**Authors:** Ruona Shi, Xue Shi, Dajiang Qin, Shibing Tang, Michiel Vermeulen, Xiaofei Zhang

**Affiliations:** 1 CAS Key Laboratory of Regenerative Biology, Guangdong Provincial Key Laboratory of Stem Cell and Regenerative Medicine, Hefei Institute of Stem Cell and Regenerative Medicine, Center for Cell Lineage and Development, Guangzhou Institutes of Biomedicine and Health, Chinese Academy of Sciences, Guangzhou, 510530 China; 2 University of Chinese Academy of Sciences, Beijing, 100049 China; 3 Center for Chemical Biology and Drug Discovery, Guangzhou Institutes of Biomedicine and Health, Chinese Academy of Sciences, Guangzhou, 510530 China; 4 Department of Molecular Biology, Faculty of Science, Radboud Institute for Molecular Life Sciences, Oncode Institute, Radboud University Nijmegen, Nijmegen, the Netherlands; 5 Center for Cell Lineage and Atlas (CCLA), Bioland Laboratory, Guangzhou Regenerative Medicine and Health Guangdong Laboratory, Guangzhou, 510530 China

**Keywords:** Linear ubiquitination, OTULIN, SNX27, NF-κB, Interaction, Localisation

## Abstract

**Background:**

Linear ubiquitination is a novel type of ubiquitination that plays important physiological roles in signalling pathways such as tumour necrosis factor (TNF) signalling. However, little is known about the regulatory mechanisms of linear ubiquitination, except the well-described enzymatic regulators E3 ligase linear ubiquitin chain assembly complex (LUBAC) and deubiquitinase OTULIN.

**Results:**

Previously, we identified SNX27, a member of the sorting nexin family protein, as a selective linear ubiquitin chain interactor in mass spectrometry-based ubiquitin interaction screening. Here, we demonstrated that the interaction between the linear ubiquitin chain and SNX27 is mediated by the OTULIN. Furthermore, we found that SNX27 inhibits LUBAC-mediated linear ubiquitin chain formation and TNFα-induced signalling activation. Mechanistic studies showed that, upon TNFα stimulation, OTULIN-SNX27 is localised to membrane-associated TNF receptor complex, where OTULIN deubiquitinates the linear polyubiquitin chain that formed by the LUBAC complex. Significantly, chemical inhibition of SNX27-retromer translocation by cholera toxin inhibits OTULIN membrane localization.

**Conclusions:**

In conclusion, our study demonstrated that SNX27 inhibits TNFα induced NF-κB signalling activation via facilitating OTULIN to localize to TNF receptor complex.

**Supplementary Information:**

The online version contains supplementary material available at 10.1186/s13578-021-00659-5.

## Background

Linear ubiquitination is one of the eight homotypic polyubiquitination linkages that forms polyubiquitin chains via continual conjugation of the C-terminal of the distal ubiquitin and the N-terminal of the proximal ubiquitin [1]. To-date, the mammalian linear ubiquitin chain assembly complex (LUBAC), which comprises HOIL-1, SHARPIN and HOIP, is the only E3 complex that catalyses linear ubiquitin (Met1) linkage formation, while OTULIN is the specific deubiquitinase that only hydrolyses Met1 linkage [2]. The molecular functions of linear polyubiquitin chain is achieved via serving as a protein–protein interaction platform to recruit signalling activators and therefore transmit signals from the receptor to downstream effectors [3]. Multiple studies have demonstrated that linear ubiquitination modifies key receptors and adaptor proteins and participates in inflammatory signalling activation, such as TNFα signalling [4]. Therefore, it is unsurprising that mutations in LUBAC and OTULIN have been found in multiple human inflammatory diseases [5–7].

Proper regulation of membrane protein recycling is crucial for cellular homoeostasis, and dysregulation of protein membrane recycling leads to cellular dysfunction [8]. Many proteins, including Sorting Nexins (SNXs), are known to be involved in this process. All SNXs have a Phox (PX)-domain, a phospholipid-binding domain, which facilitates binding to phosphatidylinositol 3-phosphate enriched early endosomes [9, 10]. Physiologically, SNXs are a component of the retromer, a protein complex that responsible for the recycling of transmembrane receptors from endosomes to the trans-Golgi network. In addition, some SNXs also participate in endocytosis and protein degradation [9, 10]. Although SNXs have common functions in recycling, they regulate different receptors. This cargo specificity is mainly mediated by SNXs-specific protein interacting domains. For example, SNX27 differs from other SNXs in that it bears a unique PDZ domain, which is normally found in proteins related to postsynaptic density of excitatory neuronal synapses [11]. The PDZ is the major cargo recognition domain of SNX27, which binds to a conserved motif X-S/T-X-Φ [12]. Previous studies have showed that SNX27 involves in the recycling of multiple receptors, such as NMDAR, AMPAR, GRP17 and plays important roles in neuronal functions [13, 14].

In our previous ubiquitin signalling interactors screening dataset, we found that SNX27 is specifically enriched by Met1 linkage [15]. Since Met1 linkage plays essential roles in TNFα-induced regulation of NF-κB signalling [3], we hypothesised that SNX27 may affect NF-κB signalling by regulating Met1 linkage formation. Here, we show that the interaction between SNX27 and Met1 linkage is mediated by OTULIN, consistent with the recent reported interaction of SNX27 and OTULIN [16]. Significantly, we found that SNX27 inhibits the activation of TNFα-induced NF-κB signalling. Mechanistic studies showed that SNX27 regulates the TNFα receptor complex associated OTULIN and linear ubiquitination. In addition, we provided evidence that the effects of SNX27 on NF-κB signalling depend on the linear ubiquitin chain formation. Finally, we inhibited the plasma membrane trafficking of SNX27-retromer using chemical compound, and we found that this inhibition prevents the translocation of OTULIN to TNF receptor complex.

## Results

### Enrichment of SNX27 by Met1 is mediated via OTULIN

**Fig. 1:**
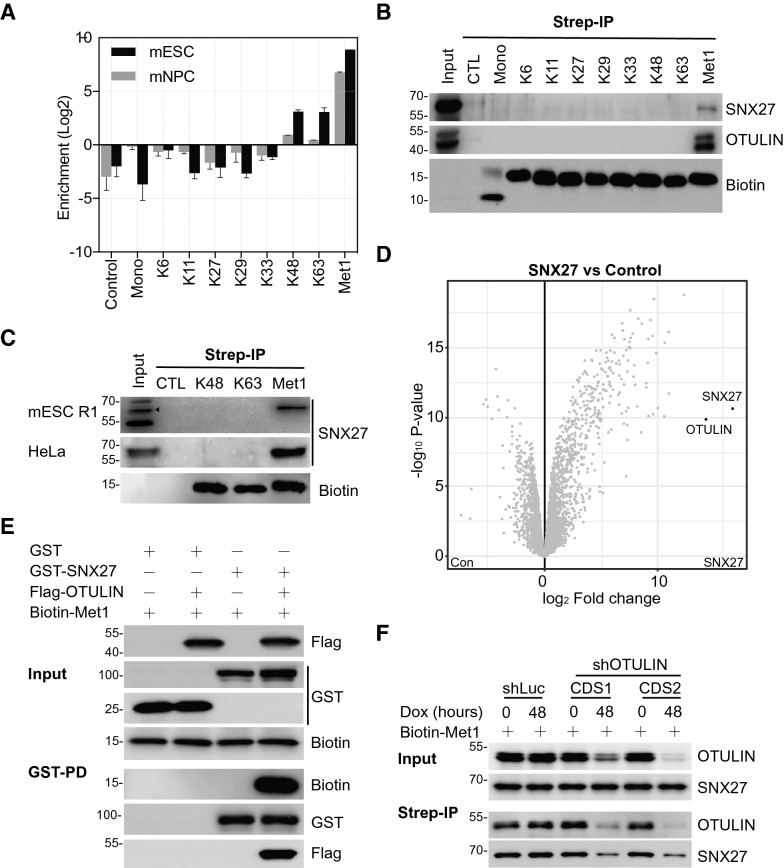
SNX27 interacts with Met1 via OTULIN. **A** SNX27 is selectively enriched by Met1 linkage. Bar plot showed enrichment of SNX27 by di-ubiquitin linkages in mESC and NPC. The raw data was obtained from previous publication [15]. **B** Streptavidin based immunoprecipitation assay to validate the interaction between SNX27 and di-ubiquitin linkages. Biotin-tagged di-ubiquitin coupled to streptavidin beads were incubated with HeLa whole cell lysate. SNX27 and OTULIN antibodies were used to detect the interaction after immunoblotting. Immunoblotting was performed at least twice, and one representative figure was shown. **C** Streptavidin based immunoprecipitation assay to validate the interaction between SNX27 and Met1 linkage in HeLa and mES cells. Biotin-tagged K48, K63 and Met1 di-ubiquitin coupled to streptavidin beads were incubated with HeLa or mES whole cell lysate. SNX27 antibody were used to detect the interaction after immunoblotting. Black triangle indicates specific signal. **D** Interaction proteomics analysis of SNX27. HeLa cells expressing control vector or Flag-SNX27 were lysed and incubated with Flag-M2 beads, after which protein on-bead digestion and peptide desalting were performed before mass spectrometry analysis. Data were analysed using DEP package as described [17]. OTULIN and SNX27 are highlighted. Full dataset is available in Additional file 6: Table S1. **E** OTULIN mediates the interaction of SNX27 and Met1 linkage in vitro. Bacterial recombinant expressed GST or GST-SNX27 were incubated with Met1 di-ubiquitin in the presence or absence of Flag-OTULIN. Indicated antibodies were used to detect the protein in input and pull-down samples. **F** OTULIN is required for SNX27 and Met1 linkage interaction in vivo. HeLa cells with inducible shRNA-mediated knockdown for luciferase or OTULIN were used to exam the interaction between SNX27 and Met1 linkage. Cells were treated with doxycycline for indicated time. Immunoprecipitation and immunoblotting assays were performed as B

The proper interpretation of linkage specific ubiquitination is mediated by ubiquitin-interacting proteins. To explore linkage-selective interactions, we recently developed the Ubiquitin Affinity Enrichment Mass Spectrometry (UbIA-MS) workflow to study all homotypic ubiquitin interactions under nearly physiological conditions [15, 17]. In our interaction dataset, we noticed that SNX27 is significantly enriched by Met1 linkage, and to a lesser extent by K48 and K63 linkages, in both embryonic stem cells and neural progenitor cells (Fig. 1A). We first validated this interaction using biotin-labelled di-ubiquitin incubated with whole cell lysates. As shown in Fig. 1B, SNX27 was detectable only in the Met1 linkage, but not in other linkages in the immunoprecipitated samples. The interaction between OTULIN and Met1 linkages was served as a positive control [18]. In addition, we validated this interaction in mouse embryonic stem cells (Fig. 1C). However, the bacterial-expressed SNX27 had no obvious interaction with any ubiquitin linkages (Additional file 1: Figure S1A), suggesting that the interaction between SNX27 and Met1 linkage might be indirect.

To identify potential adaptor proteins that link SNX27 and Met1 linkage, we analysed the SNX27 interactome using mass spectrometry (MS)-based proteomics whereby immunoprecipitation was coupled with MS to identify SNX27 interactors using Flag-resin. In total, compare to control cells, we identified 395 significant interactors with a threshold of fold-change > 2 and adjusted p < 0.05 (Fig. 1D and Additional file 6: Table S1). Gene ontology (GO) and Kyoto Encyclopedia of Genes and Genomes (KEGG) enrichment analyses showed that the significant interactors were highly enriched for regulation of GTPase activity, retrograde transport, endocytosis and endosomal transport (Additional file 1: Figure S1B and Figure S1C), which are consistent with reported SNX27 functions [19, 20]. Interestingly, we noticed a robust enrichment of NOD-like receptor and MAPK signalling pathways. We reasoned that SNX27 might also participate in these two pathways. Further studies are needed to investigate the detailed information about whether and how SNX27 regulates NOD-like receptor and MAPK signalling pathways.

Among all the significant interactors, we identified multiple known SNX27 interactors (Additional file 6: Table S1), such as FAM21A, FAM21C and VPS26B, indicating that our interaction screening was reliable [12, 21]. As highlighted in Fig. 1D, OTULIN, the only known Met1 specific deubiquitinase [22], is highly enriched by SNX27, consistent with a recent report from Stangl et al. [16]. Because the SNX27 and OTULIN antibodies are not suitable for endogenous immunoprecipitation, we alternatively showed that endogenous SNX27 and OTULIN interact with ectopic expressed OTULIN and SNX27 (Additional file 2: Figure S2A–S2C). Using recombinantly expressed proteins, we demonstrated that SNX27 directly interacts with OTULIN (Additional file 2: Figure S2D). To further study the interaction between OTULIN and SNX27, we constituted the interaction assay using constructs containing deletions and mutations of SNX27 and OTULIN. We constructed single mutation of the last 6 amino acids (CEETSL) of OTULIN, which contains the PDZ binding motif X-S/T–X–Φ (X represents any amino acid, Φ represents hydrophobic amino acid). Immunoprecipitation in 293T cells and in vitro pull-down assays using bacterial recombinant proteins indicated that the direct interaction between SNX27 and OTULIN was mediated by the PDZ domain of SNX27 and the last 6 amino acids of OTULIN (Additional file 2: Figure S2E–G), respectively.

Because OTULIN is known to be a strong interactor of Met1 linkage (Fig. 1B) [18], we hypothesized that OTULIN might be the adaptor protein for SNX27 and Met1 interaction. To test this idea, we performed an in vitro pull-down assay with SNX27 and Met1 using recombinant proteins, in the absence or presence of OTULIN. In line with our hypothesis, we found that biotin tagged Met1 was precipitated only when OTULIN was added into the interaction system together with SNX27 (Fig. 1E, lane 4 vs. lane 3). To gain insight into the mechanism of endogenous SNX27 and Met1 interaction, we constructed two inducible OTULIN knockdown cell lines. As shown in Fig. 1F, we found that the interaction between endogenous SNX27 and Met1 linkage was impaired when endogenous OTULIN expression was downregulated by inducible shRNAs. As a control, shRNA targets luciferase has no effect on SNX27 and Met1 interaction. Taken together, we showed that SNX27 is linked to Met1 linkage via the interaction with OTULIN.

### SNX27 inhibits TNFα-induced NF-κB signalling activation

**Fig. 2:**
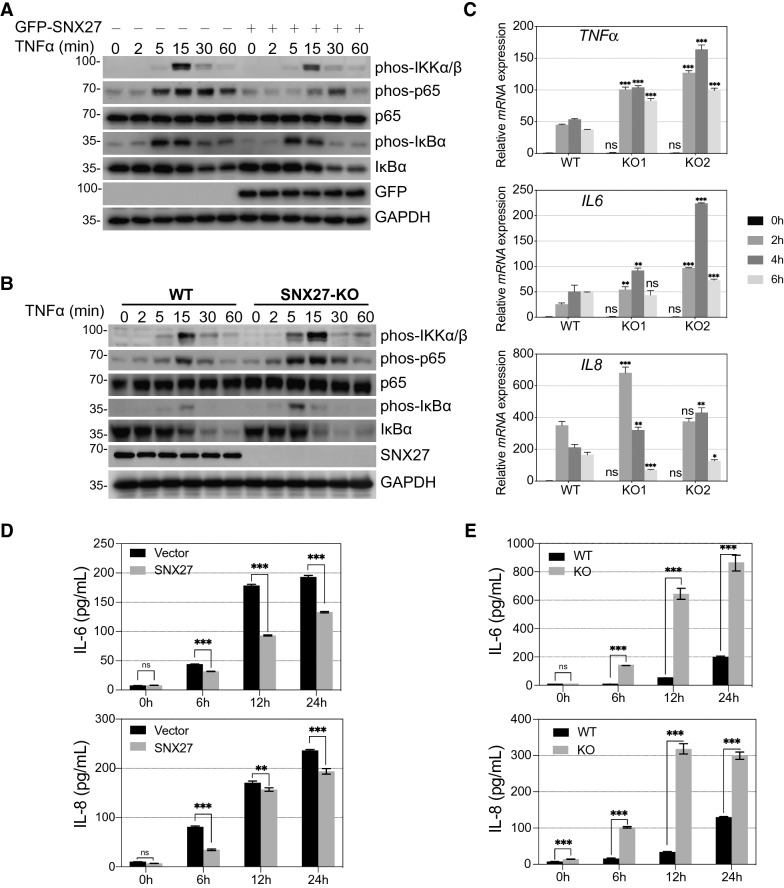
SNX27 negatively regulates TNFα-induced NF-κB signalling activation. **A** SNX27 overexpression inhibits TNFα-induced NF-κB signalling activation. HeLa cells stably expressing control vector or SNX27 were treated with TNFα for indicated time points. Phosphorylated IκBα, IKKα/β and p65 were used to exam TNFα-induced NF-κB signalling activation. The relative protein expression level that normalised to loading control was calculated by ImageJ and labelled below each blot. The value of time 0 of control cells was set as 1. Immunoblotting was performed at least twice, and one representative figure was shown. **B**, **C** Knockout of SNX27 potentiates TNFα-induced NF-κB signalling activation. HeLa cells with CRISPR/Cas9 mediated SNX27 knockout were treated with TNFα for indicated time points. Immunoblotting was performed as **A** (**B**) to check the phosphorylation of IκBα, IKKα/β and p65. Immunoblotting was performed and the relative protein expression was calculated as **A**. Quantitative real-time PCR was used to check the expression of target genes of TNFα signal (**C**). Significant differences compared to control were calculated using multiple t-tests. The graphs showed mean ± SD, n = 3. ns indicates not significant; * indicates p < 0.05, ** indicates p < 0.01; *** indicates p < 0.001. **D**, **E** SNX27 negatively regulates TNFα-induced secretion of IL-6 and IL-8. HeLa cells with SNX27 overexpression (**D**) or knockout (**E**) were treated with TNFα for indicated time points. Cell medium was collected and then the secreted IL-6 and IL-8 were quantified by ELISA kit as described in "Methods". Significant differences were calculated as described in **C**

OTULIN has been shown to specifically cleave Met1 linkage. In addition, this deubiquitinase participates in NF-κB signalling activation by regulating the abundance of receptor associated linear polyubiquitin chains formed by the LUBAC complex [23]. To study whether the interaction of SNX27, OTULIN and Met1 linkage had any impact on NF-κB signalling activation, we first analysed TNFα induced NF-κB signalling activation in cells stably overexpressing SNX27 by checking the phosphorylation of key NF-κB signalling regulators. In control cells, TNFα induced transient upregulation of phosphorylated IκBα, IKKα/β and p65, indicating the activation of NF-κB signalling. In contrast, SNX27 overexpression inhibited TNFα phosphorylation of IκBα, IKKα/β and p65 (Fig. 2A, GFP-SNX27 expressing cells). Furthermore, transcriptional expression of *TNFα*, *IL6* and *IL8* induced by TNFα were significantly inhibited by SNX27 overexpression (Additional file 3: Figure S3A and S3B). These observations indicated that SNX27 negatively regulated TNFα-induced phosphorylation of key NF-κB signalling regulators and downstream target genes. To further study the effects of SNX27 on NF-κB signalling, we constructed SNX27 knockdown and knockout cells and examined TNFα-induced phosphorylation of key regulators and downstream genes activation. The effects of CRISPR/Cas9 mediated SNX27 knockout was confirmed by genome DNA sequencing and immunoblotting. As shown in Fig. 2B and S3C, both SNX27 knockdown and knockout potentiated TNFα-induced phosphorylation of IκBα, IKKα/β and p65. Consistently, we showed that TNFα-induced target genes expression were significantly upregulated in SNX27 knockout cells (Fig. 2C). Furthermore, we showed that the phosphorylation of IκBα, IKKα/β and p65 were upregulated in SNX27 knockout mouse embryonic fibroblast (MEF) cells compared to control cells (Additional file 3: Figure S3D). In addition, we detected the amount of secreted IL-6 and IL-8 in SNX27 dysregulated cells using enzyme-linked immunosorbent assay. As shown in Fig. 2D and 2E, overexpression of SNX27 inhibited TNFα induced secretion of IL-6 and IL-8, while knockout of SNX27 potentiated the secretion.

### SNX27 counteracts linear polyubiquitin chain formation to inhibit the NF-κB signalling pathway

**Fig. 3:**
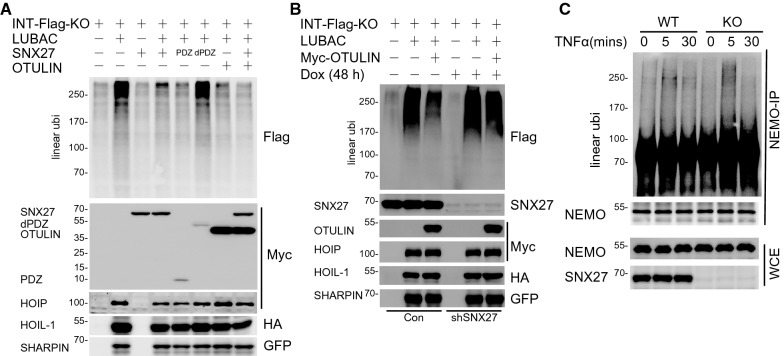
SNX27 inhibits LUBAC-mediated linear polyubiquitination. **A** SNX27 inhibits LUBAC-mediated linear polyubiquitin chain formation. HEK293T cells transfected with indicated plasmids were lysed and blotted with indicated antibodies. Linear polyubiquitin chain formation was detected using Flag antibody. INT-Flag-KO represent a lysine-deficient ubiquitin plasmid with an internal Flag tag [26]. Immunoblotting was performed at least twice, and one representative figure was shown. **B** Knockdown of SNX27 potentiates LUBAC mediated linear polyubiquitin chain formation. HEK293T cells stably expressing inducible shRNA for SNX27 were transfected with indicated plasmids, doxycycline was added for 36 h to induce knockdown of SNX27 after transfection. Immunoblotting was performed as **A**. **C** Knockout of SNX27 potentiates TNFα induced linear ubiquitination of NEMO. Cells were treated with TNFα at indicated time points and lysed in denature buffer with 1% SDS. Diluted cell lysates (0.1% SDS) were used for immunoprecipitation assay with NEMO antibody. Linear ubiquitin chain antibody was used to detect the linear ubiquitination of NEMO. Immunoblotting was performed as **A**

Since the formation of linear polyubiquitin chains is vital for TNFα-induced NF-κB signalling activation and given that OTULIN is the specific deubiquitinase for linear polyubiquitin chains [24, 25], we next sought to investigate whether SNX27 had any impact on Met1 linkage formation. Previous report has demonstrated that SNX27 has no impact on OTULIN cleavage activity for Met1 linkage in vitro [16]. Therefore, we decided to study linear polyubiquitin chain formation in vivo using a ubiquitin mutant with all lysine residues mutated to arginine residues and having an internal Flag tag (INT-Flag-KO). This lysine deficient ubiquitin has been shown to form Met1 linkage preferably and is a useful tool to study linear ubiquitination [26]. As shown in Fig. 3A, linear polyubiquitin chain formation was strongly induced by LUBAC transfection (Fig. 3A, lane2 vs. lane 1), and inhibited by the co-transfection of OTULIN (Fig. 3A, lane 7 vs. lane 2). Of importance, we observed that linear polyubiquitin chain formation was inhibited when cells were co-transfected with SNX27 (Fig. 3A, lane 4 vs. lane 2). Significantly, the inhibition effects of SNX27 depended on the PDZ domain, as SNX27 lacking PDZ had less inhibitory effect on linear polyubiquitin chain formation (Fig. 3A, lane 6 vs. lane 4). In fact, the PDZ domain of SNX27 alone was enough to inhibit LUBAC-induced Met1 linkage formation (Fig. 3A, lane 5 vs. lane 2). This result suggested that SNX27 inhibits the linear polyubiquitin chain formation, and that this inhibition depends on the PDZ domain in vivo. This inhibitory effect of SNX27 on Met1 linkage formation was further supported by the finding that the LUBAC complex-induced linear polyubiquitin chain formation was potentiated in SNX27 knockdown cells (Fig. 3B). Accordingly, we examined whether substrates linear polyubiquitination catalysed by LUBAC was inhibited by SNX27 expression. As shown in Additional file 4: Figure S4A, LUBAC-mediated linear polyubiquitination of NEMO, a well-known Met1 linkage substrate, was inhibited by SNX27 overexpression. More importantly, we showed that TNFα induced linear polyubiquitination of endogenous NEMO was increased in SNX27 knockout cells (Fig. 3C).

This finding, together with the observation that SNX27 inhibited TNFα-induced NF-κB signalling activation, led us to question whether the inhibitory effects of SNX27 on NF-κB signalling depended on linear polyubiquitin chain formation. To answer this, we used a recently reported LUBAC inhibitor, JTP-0819958, which targets LUBAC enzymatic activity and therefore inhibits TNFα-induced NF-κB signalling activation [27]. Consistent with the reported result, JTP-0819958 treatment mitigated TNFα-induced phosphorylation of p65 and IκBα (Additional file 4: Figure S4B). More importantly, we found that JTP-0819958 treatment abolished the increase of phosphorylated p65 and IκBα in SNX27 knockout cells upon TNFα-stimulation. This result indicated that the effects of SNX27 on TNFα-induced signalling activation depends on linear ubiquitin chain formation. Thus, we provide the evidence that LUBAC-catalysed Met1 linkage is important for the negative regulation of TNFα-induced NF-κB signalling activation by SNX27.

### TNFα-induced membrane localisation of OTULIN is regulated by SNX27

**Fig. 4:**
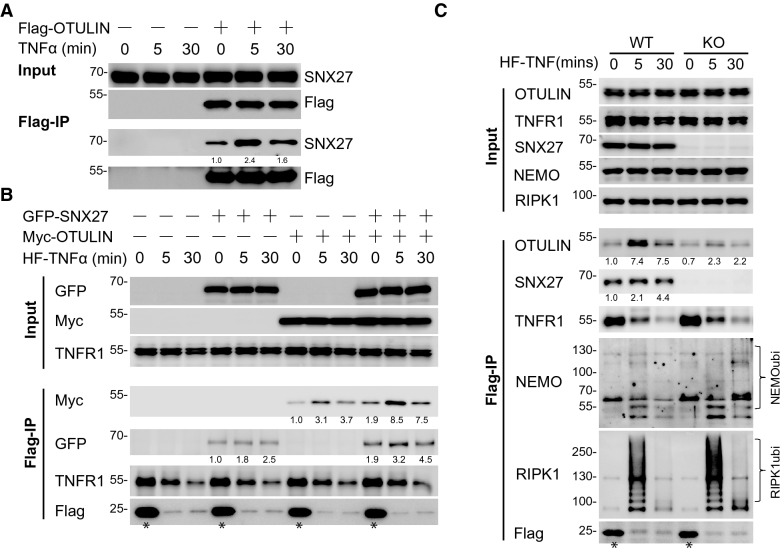
SNX27 facilitates TNFα-induced receptor associated OTULIN. **A** TNFα stimulation potentiates the interaction of SNX27 and OTULIN. HeLa cells transfected with control vector or Flag-OTULIN virus were treated with TNFα and immunoprecipitated using Flag-resin. SNX27 antibody was used to detect the interaction. The relative enrichment of SNX27 that normalised to its corresponding input was quantified by ImageJ and labelled below each blot. The value of time 0 of OTULIN overexpression cells was set as 1. Immunoblotting was performed at least twice, and one representative figure was shown. **B** SNX27 facilitates TNFα-induced receptor complex associated OTULIN. HeLa cells were transfected with control vector, OTULIN and/or SNX27 viruses. Cells were treated with Flag-tagged TNFα for indicate time points before harvest. Immunoprecipitation and immunoblotting were performed as **A**. Asterisks indicate the amount of Flag-TNFα in control condition was much higher than in stimulation conditions because most Flag-TNFα was washed away in stimulation conditions. The relative enrichment of SNX27 and OTULIN that normalised to immunoprecipitated TNFR1 was quantified by ImageJ and labelled below each blot. The value of time 0 of SNX27 and OTULIN overexpressed cells was set as 1. **C** Depletion of SNX27 impairs TNFα-induced receptor complex associated OTULIN. HeLa parental and SNX27 knockout cells were treated with Flag-tagged TNFα for indicate time points. Immunoprecipitation and immunoblotting were performed as Fig. 4A. The relative enrichment of SNX27 and OTULIN that normalised to immunoprecipitated TNFR1 was quantified by ImageJ and labelled below each blot. The value of time 0 of each immunoprecipitated protein was set as 1. Asterisks were used as indicated in **B**

To determine the underlying mechanisms by which SNX27 regulates linear polyubiquitin chain formation by interacting with OUTLIN, we first investigated the interaction of SNX27 and OTULIN upon TNFα-stimulation. As shown in Fig. 4A, the interaction between endogenous SNX27 and OTULIN was upregulated when cells were treated with TNFα for 5 min. This increment returned to steady-state levels when cells were treated for 30 min. Because TNFα-induced membrane receptor-associated OTULIN was similarly transiently induced as observed with the interaction dynamics between SNX27 and OTULIN [28], we tested whether TNFα-induced membrane receptor-related OTULIN was regulated by SNX27. To accomplish this, we used Flag-tagged TNFα to stimulate cells and to immunoprecipitate TNFα receptor complex-related proteins. We used equally concentration of Flag-tagged TNFα for all conditions (Fig. 4B). However, it should be noted that for stimulated time points, unbounded Flag-tagged TNFα was washed away. While for time point 0, Flag-tagged TNFα was directly added into cell lysates. Therefore, the immunoprecipitated TNFα in time point 0 (indicated with asterisk) was much higher than stimulated conditions. These differences also explained why we immunoprecipitated less TNFR1 in TNFα treated cells than time point 0. In line with previous report [28], we observed increased association of OTULIN with TNFα receptor complex upon TNFα stimulation (Fig. 4B, lanes 7–9, normalized to immunoprecipitated TNFR1), thought the immunoprecipitated TNFα was much less than control. Importantly, we observed an increase in TNFα receptor complex associated OTULIN in cells with SNX27 overexpression (Fig. 4B, lanes 10–12 vs. lanes 7–9). To further validate this observation, we treated SNX27 knockout cells with Flag-tagged TNFα to immunoprecipitate receptor associated OTULIN. As shown in Fig. 4C, the abundance of TNFR1 associated OUTLIN was decreased in SNX27 knockout cells. In addition, we checked the abundances of TNFR1 complex associated ubiquitinated NEMO and RIPK1, which reflect TNFα induced NF-κB signalling activation [29]. As shown in Fig. 4C, we observed that TNFα stimulated and TNFR1 associated ubiquitinated RIPK1 and NEMO was potentiated in SNX27 knockout cells. This result suggested that SNX27 facilitates OTULIN to TNFR1 complex, which in turn influences the TNFR1 complex formation for signalling activation.

**Fig. 5:**
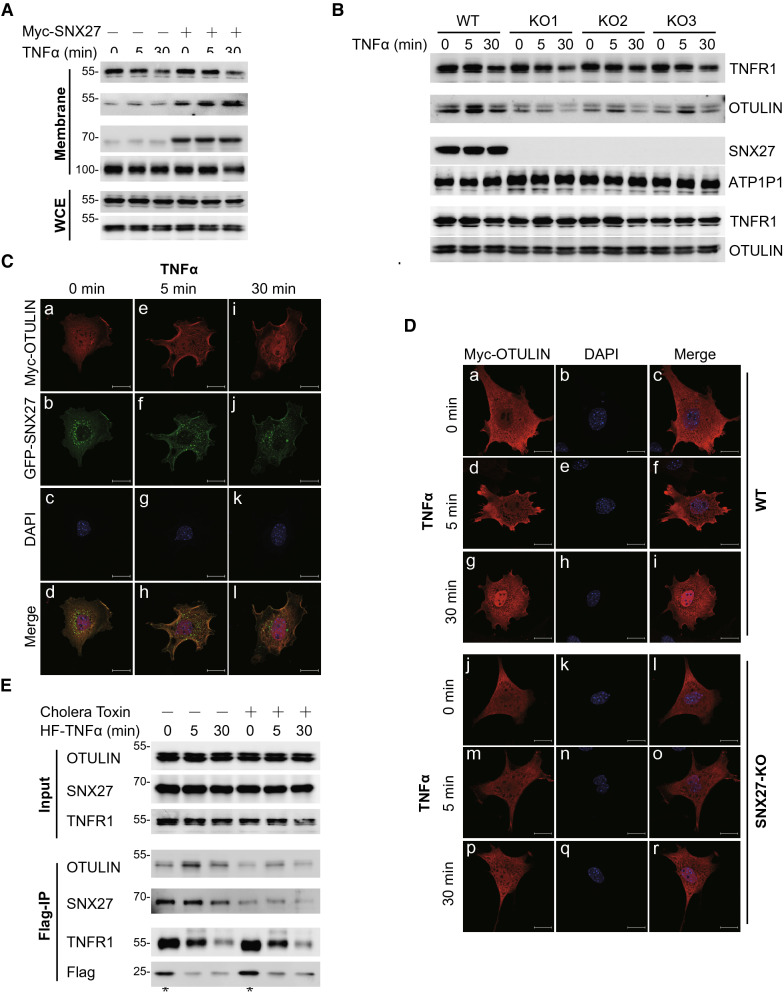
SNX27 facilitates TNFα-induced membrane-localized OTULIN. **A** and **B** SNX27 facilitates TNFα-induced membrane associated OTULIN. Membrane proteins of HeLa cells with SNX27 overexpression (**A**) or knockout (**B**) with TNFα stimulation were isolated by cell fractionation kit. Immunoblotting experiments were performed to check the membrane associated OTULIN and TNFR1. ATP1A1 was served as a membrane marker and loading control. The relative protein expression level that normalised to loading control was calculated by ImageJ and labelled below each blot. The value of time 0 of control cells was set as 1. Immunoblotting was performed at least twice, and one representative figure was shown. **C** SNX27 facilitates TNFα-induced membrane localization of OTULIN. MEF cells infected with Myc-OTULIN and GFP-SNX27 lentivirus were treated with TNFα for indicated time points and fixed for immunostaining. Myc antibody was used to detect the localization of OTULIN. Immunostaining was performed as described in "Methods". Scale bar represents 20 μm. One representative experiment was shown. **D** SNX27 depletion inhibits TNFα-induced membrane localization of OTULIN. Wild-type and SNX27 KO MEF cells were infected with Myc-OTULIN lentivirus and immunostaining assay was performed as Fig. 5C. Scale bar represents 20 μm. One representative experiment was shown. **E** Inhibition of SNX27 trafficking decrease TNFα-induced receptor associated OTULIN. HeLa cells were treated with cholera toxin for 24 h and then Flag-TNFα was added at indicated time points before harvest. Immunoprecipitation and immunoblotting were performed to detect TNFα receptor associated OTULIN. The relative enrichment of SNX27and OTULIN that normalised to immunoprecipitated TNFR1 was calculated by ImageJ and labelled below each blot. The value of time 0 without cholera toxin treatment was set as 1. Asterisks were used as indicated in Fig. 4B

The above observations suggested that SNX27 helps the association of OTULIN to TNFR1 complex. To validate this hypothesis, we first isolated the membrane proteins from SNX27 dysregulated cells using a commercial cell fractionation kit. Consistent with previous reported TNFα stimulation induces internalization of TNFR1 [30, 31], we also found that membrane associated TNFR1 was decreased upon TNFα treatment. However, dysregulation of SNX27 has no obvious influence on membrane associated TNFR1 (Fig. 5A and 5B). Interestingly, we observed the membrane related OTULIN was increased in cells with SNX27 overexpression (Fig. 5A). In contrast, the membrane related OTULIN was decreased in three independent SNX27 knockout cells (Fig. 5B). These results clearly indicated that SNX27 positively regulates TNFα-induced receptor complex associated OTULIN localisation. To further validate our hypothesis, we used immunostaining to check the cellular localization of OTULIN. We showed that the extent of membrane localization of OTULIN was relatively small, even with TNFα stimulation (Additional file 5: Figure S5A), while SNX27 alone showed punctate cytoplasmic localization (Additional file 5: Figure S5B). In comparison, we observed increased membrane localization of OTULIN when co-expressed with SNX27 upon TNFα stimulation (Additional file 5: Figure S5C). We also validated this observation in mouse embryonic fibroblast cells. As shown in Fig. 5C, we showed that the co-localization of SNX27 and OTULIN was increased upon TNFα stimulation in MEF cells with SNX27 overexpression. In addition, we showed that OTULIN had no obvious membrane localization in SNX27 knockout MEF cells even with TNFα treatment, compared with wild-type MEF cells (Fig. 5D, WT vs. SNX27-KO). Finally, we tested the effects of cholera toxin, a compound that induces the phosphorylation of SNX27 and inhibits SNX27-mediated cargo trafficking to the plasma membrane [32], on TNFα-induced membrane localization of OTULIN. As shown in Fig. 5E, cholera toxin strongly inhibited SNX27-mediated TNFα-stimulation mediated OTULIN membrane localization. As a control, the interaction between TNFα and TNFR1 was not obviously affected by retromer inhibition. Thus, we concluded that SNX27 facilitates TNFα-induced membrane localization of OTULIN.

## Discussion

Retromer-mediated protein trafficking has proven to be an efficient way for cell to regulate protein localization and therefore regulate protein functions [8]. SNX27 is a retromer protein that regulates protein trafficking from early endosome to the plasma membrane [33]. Previous study has shown that OTULIN negatively regulates SNX27 dependent cargo loading [16]. However, the effects of SNX27 on OTULIN’s trafficking has not been studied. In this study, we combined immunoprecipitation, immunostaining and cell fractionation assay to show that SNX27 potentiates the localization of OTULIN to TNF receptor complex. The observed inhibitory effects of cholera toxin on membrane associated OTULIN strengthen our conclusion, because cholera toxin inhibits SNX27 mediated plasma membrane trafficking of cargo proteins [32]. Therefore, we speculated that when cells are undergoing TNFα stimulation, the interaction between OTULIN and SNX27 will be transiently increased, which subsequently decreases the loading of other SNX27 dependent cargo proteins [16]. In the meantime, SNX27 transports OTULIN to the membrane associated TNFα receptor complex, where OTULIN limits LUBAC induced linear polyubiquitination. Therefore, we concluded that the interaction between OTULIN and SNX27 has mutual effects on each other.

The LUBAC and OTULIN are the enzymes that maintain the homeostasis of the linear polyubiquitination in cells. And these proteins are shown to play essential roles in TNFα induced NF-κB signalling activation, and mutations of them have been found in multiple inflammatory diseases [4–7]. It, therefore, is necessary to study how cell regulates the activity of these enzymes, and the consequence of these regulations on signalling transduction. In this study, we found that SNX27 negatively regulates TNFα induced NF-κB signalling activation via regulating the localization of OTULIN. We showed that SNX27 negatively regulates the linear ubiquitination of NEMO, a key mediator of NF-κB signalling pathway [18]. In addition, we found that the association of ubiquitinated NEMO and RIPK1 to TNFR1 complex was negatively regulated by SNX27, indicating the inhibitory effects of SNX27 on TNFα signalling. Furthermore, our results of phosphorylation of key regulators (p65, IκBα and IKKα/β) and expression of target genes and proteins clearly indicated that SNX27 negatively regulated TNFα-induced NF-κB signalling activation.

Previous study from *Wang *et al*.* showed that the depletion of SNX27 causes severe neuronal deficits in mice, and these mice phenocopying Down’s syndrome brains [13]. Also, Down’s syndrome patients frequently display increased incidence of chronic inflammatory diseases [34]. Therefore, it would be attractive to exam the activity of NF-κB signalling and level of linear ubiquitination in Down’s syndrome patient-derived samples, such as induced pluripotent stem cells. Given the factor that SNX27 was immunoprecipitated by TNFα at steady status, it would be interesting to investigate whether SNX27 interferes the binding of TNFR1 to other downstream proteins. Another interesting study would be to exam the effects of SNX27 on NOD-like receptor and MAPK signalling pathways, as we noticed that interactors of SNX27 are enriched for these two terms (Additional file 1: Figure S1C). Finally, our interactome results provide a list of candidates to study the potential functions of SNX27 in the future (Additional file 6: Table S1, Figure S1B and S1C).

## Conclusions

In summary, we showed that the interaction of SNX27 and OTULIN potentiates the translocation of OTULIN to TNFα receptor complex. The relocated OTULIN subsequently restricts LUBAC complex catalysed linear polyubiquitination and therefore inhibits TNFα induced NF-κB signalling activation. We believe our observations provide a novel mechanism of how SNX27-mediated protein trafficking affects linear ubiquitination and signalling activation.

## Methods

### Cell culture and plasmids

HEK293T, HeLa (purchased from ATCC) were cultured in Dulbecco’s Modified Eagle’s Medium (DMEM, Hyclone, SH30033.01) supplemented with 10% Fetal bovine serum (FBS, Hyclone, SV30160.03) and 1 × Penicillin/Streptomycin (Hyclone, SV30010-10). MEF cells from *Snx27* knockout mice (Gempharmatech, China) were culture in same medium as HEK293T cells. All cells including the established stable cell lines were routinely test for mycoplasma using MycoAlert Mycoplasma Detection Kit (Lonza, LT07-318). For stimulation, cells were treated with 10 ng/mL of TNFα (starved with DMEM for 6 h, 8902SF, Cell Signaling) or compounds for indicated time points. JTP-0819958 was synthesized as reported [27]. 50 μM of JTP-0819958 were added into cells 6 h before the harvest. 200 ng/mL of cholera toxin (HY-P1446, MedChemExpress) were added to cells for 48 h before the harvest.

Full-length of SNX27 and OTULIN from cDNA of HeLa and pOPINB-OTULIN (a gift from David Komander, addgene: 61464) [18] were cloned into either pLV lentivirus vector or pCR3.1 vector with indicated tag. shRNAs of luciferase (cattctatcctctagaggatg), SNX27 (gtgtgttcaatacgagtaatt,) and OTULIN (shRNA1: ccctcatctatgatgcaatat, shRNA2: ggcatcagaaccgagattaag) were cloned into an inducible lentivirus vector with tetracycline operated H1 promoter. sgRNAs (caattagatgcccgacgtcg) targets SNX27 were cloned into vector pSpCas9(BB)-2A-Puro (a gift from Feng Zhang, addgene: 48,139) [35]. All plasmids were confirmed by DNA sequencing.

### Immunoblotting and immunoprecipitation

Whole cell extracts were prepared using lysis buffer (0.5% NP40, 150 mM NaCl, 50 mM Tris pH 8.0, 10% Glycerol) with fresh added 1 × protease inhibitors (Sigma, 11697498001) for 30 min on rotation wheel at 4 °C. After centrifuged with 20,000*g* at 4 °C for 10 min, protein concentration was measured with BCA assay following instructions (Thermo, 23225). For TNFR1 complex immunoprecipitation assay, cells were starved with DMEM for 6 h and stimulated with 5 mL of 100 ng/mL HF-TNFα for indicated time points. For time point 0, HF-TNFα was directly added into cell lysates. For immunoprecipitation assay, equally amounts of supernatants were incubated with 20 μL of indicated beads (GFP-Trap Chromotek, gtma-200; Flag-resin, Sigma, A2220 and Myc-resin, Thermo, 20169) for 2 h. All these processes were performed at 4 °C or on ice. The precipitates were washed three times with lysis buffer and bound proteins were eluted by boiling with 2 × LDS loading buffer (Thermo, NP0008) at 95 °C for 5 min. Cell subcellular fractionation assay was performed according to manufacturer’s instruction (Thermo, 78840). Immunoblotting was performed using Bio-Rad mini-gel running and blotting system and imaged with Bio-Rad Chemi-Doc. Antibodies used were: SNX27 (Abcam, ab77799 and Beythl, A305-439A-T), OTULIN (Cell Signaling, 14127), Flag (Sigma, F1804), Myc (Proteintech, 16286–1-AP), HA (Sigma, H6908), GAPDH (GeneTex, GTX100118), GST (Thermo, PA1-982A), GFP (Santacruz, SC-9996 and Proteintech, 50,430–2-AP), Streptavidin-HRP (Thermo, 21,130), p65 (Cell Signaling, 8242), phosphor-p65 (Cell Signaling, 3033), IκBα (Cell Signaling, 4814), phosphor-IκBα (Cell Signaling, 2859), phosphor-IKKα/β (Cell Signaling, 2697), TNFR1 (Proteintech, 21574), linear ubiquitin (MABS451, Millipore), RIPK1 (Cell Signaling, 3493) and ATP1A1 (Abcam, ab7671).

### In vivo ubiquitination assay

NEMO ubiquitination assay was preformed either with Flag-resin or with NEMO antibody. Cells were lysed using lysis buffer supplemented with 1% SDS and 10 mM *N*-Ethylmaleimide. Lysates were then sonicated, boiled at 95 °C for 5 min and diluted to 0.1% SDS by lysis buffer. Flag immunoprecipitation assay was performed as described above. For immunoprecipitation with NEMO antibody, 1 μg of NEMO antibody (Santa Cruz, FL-419) was added into the cell lysates overnight at 4 °C. Combined Protein A/G magnetic beads (Bio-rad, 1,614,833) were added for another 1.5 h. Beads were then washed 3 times with wash cell lysis buffer and eluted with 2 × LDS loading buffer for 15 min at 42℃.

### Immunostaining

Cells grow on coverslips were washed with PBS for two times and fixed with 4% paraformaldehyde at room temperature for 30 min. Subsequently, cells were quenched with 2 mg/mL glycine in PBS, permeabilized with 0.2% Triton X-100 in PBS, washed 3 times with PBS, blocked with 10% FBS in PBS for 60 min, incubation with first and second antibodies for 60 min with 3 washes in between. All antibodies were diluted in 2% FBS in PBS. Cells were mounted using ProLong Gold Antifade Reagent with DAPI (Cell Signaling, 8961S) after 4 times PBST washes. Image visualization was using a confocal microscope (Zeiss, LSM800) or Evos FL Auto 2 (Thermo).

### Recombinant protein expression and in vitro interaction

Recombinant protein were expressed in transformed BL21 *E. coli*. cDNA of TNFα and SNX27 were cloned into pET28a vector with 6 × His-Flag tags (Flag-His-TNFα) and pGEX-5X-1 vector with GST tag, respectively. pOPINB-OUTLIN plasmid was opened by PCR and a Flag tag was added. Site-specific mutations and deletions were performed using Phanta Max (Vazyme, P505-02). Bacteria transformed with desired plasmids were grow at 37 °C until OD600 around 0.6, after which recombinant protein expression was induced with 0.5 mM IPTG (Sigma, I6578) at 16 °C overnight. Cells were lysed in PBS with 1 × protease inhibitors using high-pressure homogenizer at 1200 bar pressures (JN-Mini Pro, JNBIO Guangzhou). The cell lysates were centrifuged at 20,000*g* for 15 min and the soluble extract was aliquoted and snap frozen until further usage.

For the in vitro interaction, bait proteins were immobilized to indicated beads and washed with lysis buffer for 3 times. Then, the bacteria lysates with prey protein were incubated with the immobilized beads for 2 h at 4 °C. The precipitates were washed three times with lysis buffer and bound proteins were eluted by boiling with 2 × LDS loading buffer at 95 °C for 5 min. For validate the interaction between Met1 di-ubiquitin with endogenous SNX27, 10 μg of biotin labelled ubiquitin or di-ubiquitin were immobilized to streptavidin beads (Sigma, GE17-5113–01) then incubated with 2 mg of HeLa or ESC crude cell lysates overnight [15]. The precipitates were washed three times with lysis buffer and bound proteins were eluted by incubating with 2 × LDS loading buffer at 42 °C for 15 min to avoid the aggregation of ubiquitin. Immunoblotting was performed as described above.

### Lentivirus transduction and CRISPR/Cas9 gene editing

Lentiviral vectors were produced by transfecting HEK293T cells with the helper plasmids pCMV-VSVG, pMDLg-RRE (gag/pol) and pRSV-REV using PEI (Polysciences, 24765) as described before (Zhang et al., 2013). Briefly, cell supernatants were harvested 48 h post transfection and filter through a 0.45 μM filter. For stable infection, indicated cells were treated for 24 h with the lentivirus-containing supernatants in the presence of 8 µg/mL of polybrene (Sigma, H9268) and selected with antibiotics (puromycin, invivogen, Ant-pr-5b or hygromycin, Sigma, V900372) for at least 3 passages.

To create CRISPR/Cas9 mediated SNX27 knockout cell line, plasmid with sgRNA was transfected into HeLa cells using PEI and followed with puromycin selection for 3 passages. Cells were seeded into a very low confluence to pick up single colony after 1-week further culturing.

### Quantitative Real-time RT-PCR

HeLa cells with SNX27 overexpression or knocking out were starved for 6 h with DMEM medium. Cells were then treated with TNFα (10 ng/mL) for indicated time points. RNA extraction was performed using HP total RNA kit (R6812, OMEGA). 1 μg of RNA was retrotranscribed using HiScript III RT SuperMix for qPCR (R323, Vazyme). The quantitative real-time PCR was performed in a Biorad CFX96 system using ChamQ Universal SYBR qPCR Master Mix (Q711, Vazyme). Primers used in this study are: *SNX27-F:*catcctggaggtgaaccacg*, SNX27-R:*ctgcctccctgccatgtaaa*; TNFα-F:*ccccagggacctctctctaatcag*, TNFα-R:*ggttatctctcagctccacgcca*; IL6-F:*actcacctcttcagaacgaattg*, IL6-R:*ccatctttggaaggttcaggttg*; IL8-F:*cttggcagccttcctgattt*, IL8-R:*ttctttagcactccttggcaaaa*; GAPDH-F:*ggtggtctcctctgacttcaaca*, GAPDH-R:gttgctgtagccaaattcgttgt.* Student’s t-test was used for statistical analysis.

### Enzyme-linked immunosorbent assay (ELISA)

HeLa cells with SNX27 overexpression or knocking out were starved for 6 h with DMEM medium. Cells were then treated with TNFα (10 ng/mL) for indicated time points and medium were collected for ELISA. Secreted IL-6 and IL-8 were quantitated by human IL-6 and IL-8 ELISA kits from Proteintech according to manufacturer’s instruction (KE00007 and KE00006). Student’s t-test was used for statistical analysis.

### Immunoprecipitation for interactome identification

To identify the interaction proteins for SNX27, Flag-M2 magnetic beads (Sigma, M8823) was used to immunoprecipitate interactors from control vector and Flag-SNX27 expressing HeLa cells, respectively. The immunoprecipitation assay and on-bead digestion assay were performed as we described above and before (Zhang et al*.*, 2018). Briefly, proteins bound to Flag-M2 beads were suspended in 100 µL of elution buffer (2 M urea, 100 mM Tris 8.5, 10 mM DTT) for 20 min, incubated with 10 µL of 0.55 M iodoacetamide (Sigma, I1149) for 10 min and partially digested with 150 ng of trypsin (Promega, V5111) for 2 h. All these processes were performed at room temperature using a thermoshaker at a speed of 1200 rpm. After the incubation, the supernatant was collected in a separate tube and the beads was incubated with another 100 µL of elution buffer for 5 min at RT in a thermoshaker at 1200 rpm. The combined elutes were digested overnight at RT with additional 100 ng of trypsin. Finally, the tryptic peptides were acidified by adding 12 µL 10% TFA and desalted using homemade C18 stagetips [36].

### Mass spectrometry and data analysis

Tryptic peptides were separated using a 140 min of total data collection (100 min of 2% to 22%, 20 min 22% to 28% and 12 min of 28% to 36% gradient of acetonitrile (Thermo, 51,101) for peptide separation, following with two steps washes: 2 min of 36% to 100% and 6 min of 100% acetonitrile) with an Easy-nLC 1200 connected online to a Fusion Lumos mass spectrometer (Thermo). Scans were collected in data-dependent top-speed mode with dynamic exclusion at 90 s. Raw data were analysed using MaxQuant version 1.6.0.1 search against human Fasta database, with label free quantification and match between runs functions enabled. The output protein list was analysed and visualized using DEP package as described before [17]. GO analysis were performed using home-made R package.

## Supplementary Information

**Additional file 1: Figure S1.** SNX27 has no direct interaction with Met1 linkage, and GO and KEGG analyses of SNX27 interactors. **A** SNX27 has no interaction with any di-ubiquitin in vitro*.* Bacterial recombinantly expressed GST-SNX27 coupled to glutathione agarose were incubated with monoubiquitin and eight di-ubiquitin. Streptavidin-HRP antibody was used to detect the interaction. Immunoblotting was performed at least twice, and one representative figure was shown. **B** and **C** GO and KEGG enrichment of SNX27 interactors. Enriched GO and KEGG terms for SNX27 interactors (Table S1) were annotated on a Benjamini and Hochbery test (FDR < 0.05).

**Additional file 2: Figure S2.** The direct interaction of SNX27 and OTULIN is mediated by their PDZ domain and last 6 amino acids, respectively. **A**, **B** and **C** SNX27 interacts with OTULIN. HeLa cells with SNX27 (**A** and **B**) or OTULIN (**C**) overexpression were immunoprecipitated with GFP, Myc or Flag beads, followed immunoblotting with OTULIN (**A** and **B**) or SNX27 (**C**) antibody to detect the endogenous interaction. Immunoblotting was performed at least twice, and one representative figure was shown. **D** SNX27 direct interacts with OTULIN. Bacterial recombinantly expressed GST-SNX27 coupled to glutathione agarose were incubated with bacterial lysates expressing His-OTULIN. GST pull-down experiment was performed to study the direct interaction of SNX27 and OTULIN. Ponceau read staining and His antibody were used to detect the interaction after immunoblotting. **E** The last 6 amino acids of OTULIN interacts with SNX27. Flag tagged OUTLIN or OTULIN-d6 (without last 6 amino acids) coupled to Flag-M2 beads were incubated with bacterial lysates expression GST-SNX27. GST antibody were used to detect the interaction after immunoblotting. **F** The amino acids of OTULIN that responsible for interaction with SNX27. HEK293T cells were transfected with Myc-SNX27 and Flag-OTULIN mutations. Myc antibody was used to detect the interaction after Flag-M2 beads immunoprecipitation and immunoblotting. **G** The PDZ domain of SNX27 interacts with OTULIN. GST tagged SNX27 or SNX27-dPDZ coupled to glutathione agarose were incubated with bacterial lysates expressing Flag-OTULIN. Flag antibody were used to detect the interaction after immunoblotting.

**Additional file 3: Figure S3.** SNX27 negatively regulates TNFα-induced NF-κB signalling activation. **A** and **B** Overexpression of SNX27 inhibits TNFα-induced NF-κB signalling activation. Quantitative real-time PCR was used to check the expression of SNX27 (**A**) and target genes of TNFα signalling (**B**). Significant differences compared to control were calculated using multiple t-tests. The graphs showed mean ± SD, n = 3. ns indicates not significant; * indicates p < 0.05, ** indicates p < 0.01; *** indicates p < 0.001. **C** Knockdown of SNX27 potentiates TNFα-induced NF-κB signalling activation. HeLa cells with doxycycline induced SNX27 knockdown were treated with TNFα at indicated time points. Immunoblotting was performed as Fig. 2A to check the phosphorylation of IκBα, IKKα/β and p65. The relative protein expression level was calculated by ImageJ and labelled below each blot. The value of time 0 of control cells was set as 1. Immunoblotting was performed at least twice, and one representative figure was shown. **D** Knockout of Snx27 in MEF cells potentiates TNFα-induced NF-κB signalling activation. Immunoblotting was performed and the relative protein expression was calculated as Figure S3C.

**Additional file 4: Figure S4.** SNX27 inhibits LUBAC-mediated linear polyubiquitination and NF-κB signalling activation. **A** SNX27 inhibits linear polyubiquitination of NEMO. HEK293T cells transfected with indicated plasmids were lysed in 1% SDS buffer, followed with 10 × dilution and Flag-resin immunoprecipitation. NEMO antibody was used to detect its linear polyubiquitination. Immunoblotting was performed as Fig. 3A. Immunoblotting was performed at least twice, and one representative figure was shown. **B** LUBAC activity is required for SNX27-mediated inhibition of TNFα-induced NF-κB signalling activity. HeLa cells with CRISPR/Cas9 mediated SNX27 knockout were treated with JTP-0819958 and/or TNFα for indicated time points. Phosphorylated IκBα, IKKα/β and p65 were used to indicate TNFα-induced NF-κB signalling activity. The value of time 0 of control cells was set as 1. The relative protein expression level was calculated by ImageJ and labelled below each blot.

**Additional file 5: Figure S5.** SNX27 facilitates TNFα-induced membrane localization of OTULIN. **A**, **B** and **C** TNFα-induced membrane localization of OTULIN is potentiated by SNX27 expression. HeLa cells transfected with Myc-OTULIN (**A**), Flag-SNX27 (**B**) and Flag-SNX27 and Myc-OTULIN together (**C**) were treated with TNFα for indicated time points. Cells were fixed and stained with Flag and Myc antibodies. **D** Validation of SNX27 expression in SNX27 knockout MEF cells. Wild type and SNX27 knockout MEF cells were lysed for immunoblotting.

**Additional file 6: Table S1.** Interaction proteins of SNX27.

## Data Availability

All data and materials are available upon request.
