# Supplementary figures and images for: SNX27-driven membrane localisation of OTULIN antagonises linear ubiquitination and NF-κB signalling activation
Source: Cell Biosci. 2021 Jul 27;11:146. doi: 10.1186/s13578-021-00659-5 (PMC8314547; doi:10.1186/s13578-021-00659-5)

**A**

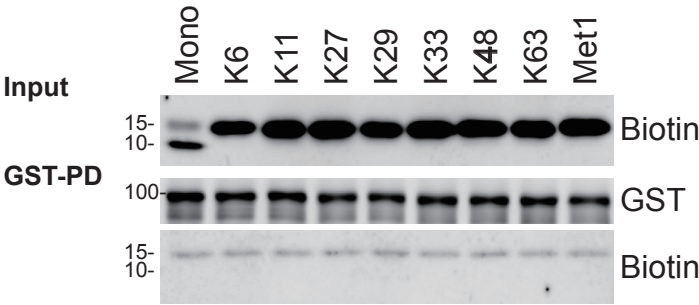

**B**

The GO enrichment analysis

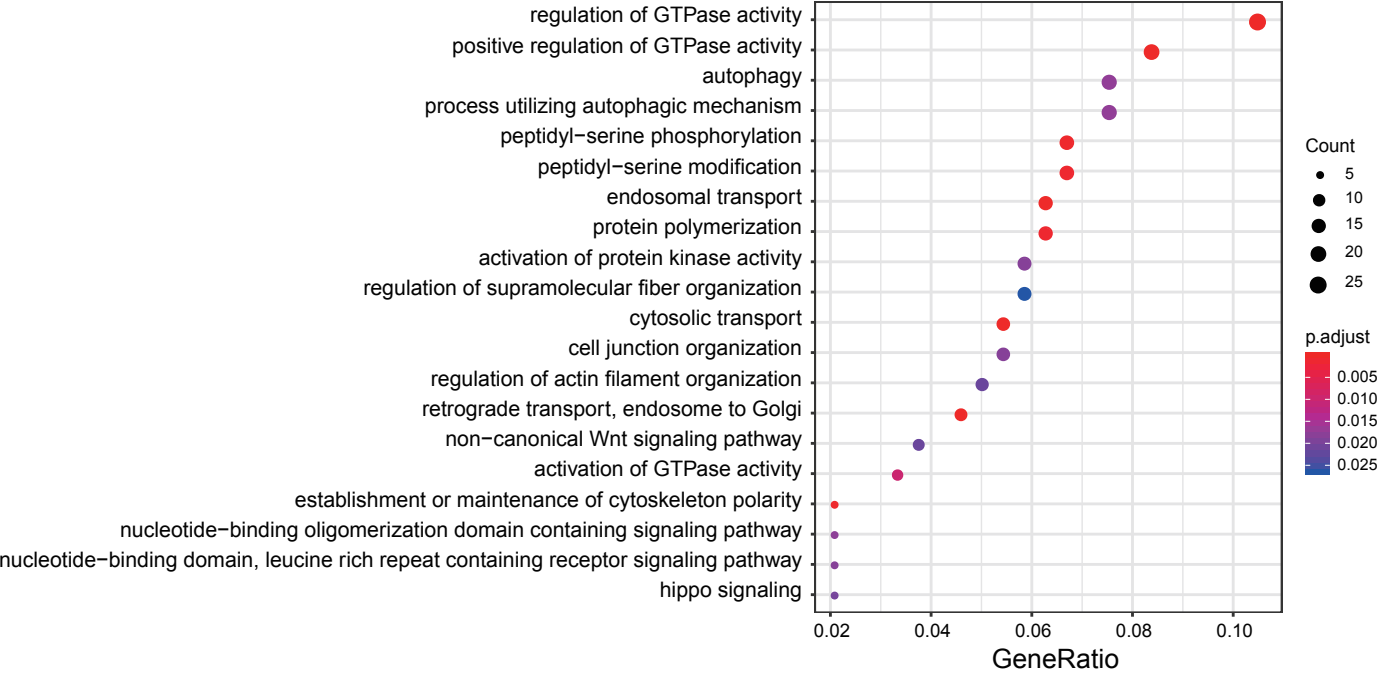

**C**

The KEGG enrichment analysis

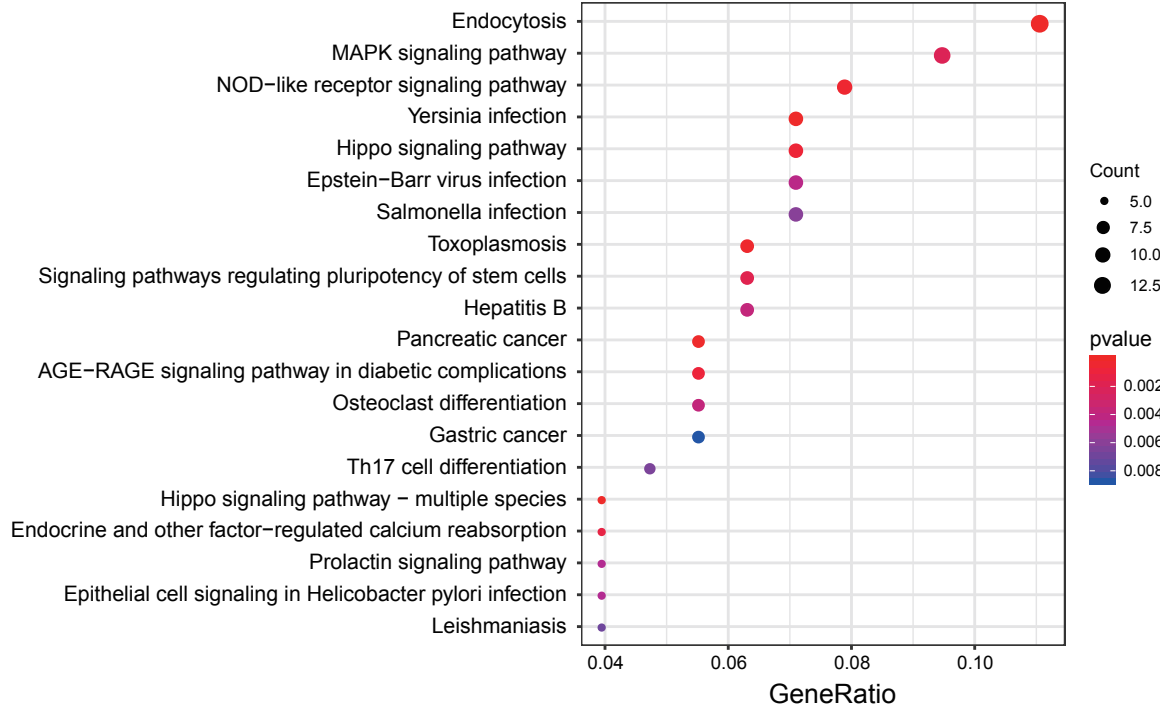

Supplement: Supplementary file 1 — Additional file 1: Figure S1. SNX27 has no direct interaction with Met1 linkage, and GO and KEGG analyses of SNX27 interactors. A SNX27 has no interaction with any di-ubiquitin in vitro. Bacterial recombinantly expressed GST-SNX27 coupled to glutathione agarose were incubated with monoubiquitin and eight di-ubiquitin. Streptavidin-HRP antibody was used to detect the interaction. Immunoblotting was performed at least twice, and one representative figure was shown. B and C GO and KEGG enrichment of SNX27 interactors. Enriched GO and KEGG terms for SNX27 interactors (Table S1) were annotated on a Benjamini and Hochbery test (FDR < 0.05). [file 13578_2021_659_MOESM1_ESM.pdf]

**A**

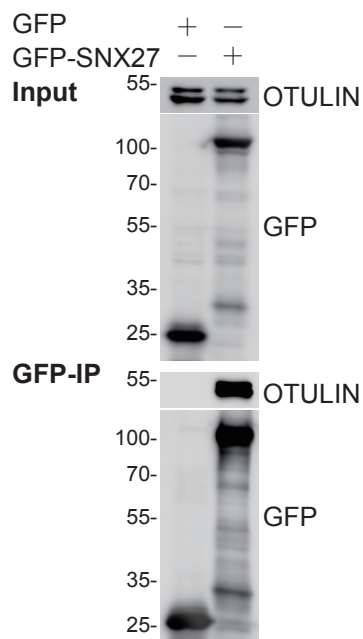

**B**

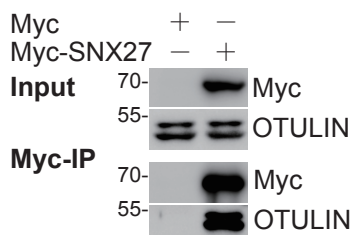

**C**

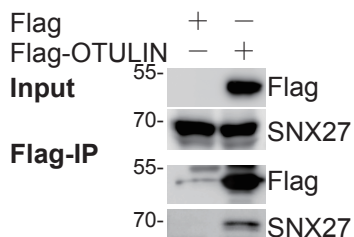

**D**

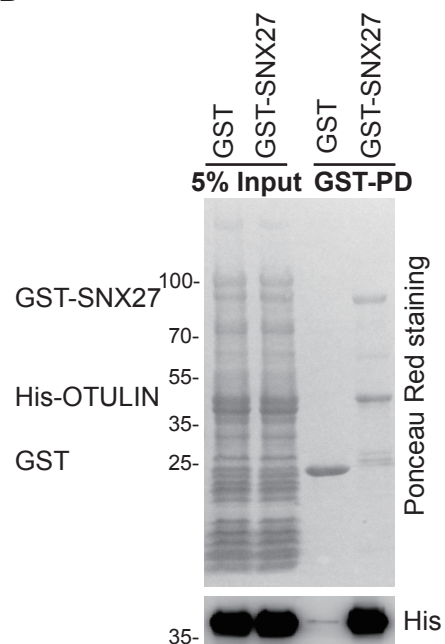

**E**

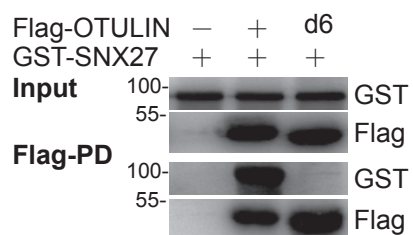

**F**

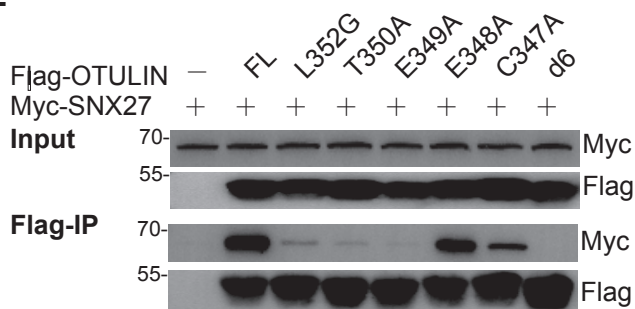

**G**

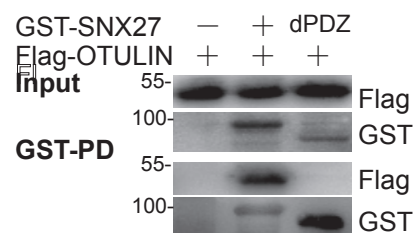

Supplement: Supplementary file 2 — Additional file 2: Figure S2. The direct interaction of SNX27 and OTULIN is mediated by their PDZ domain and last 6 amino acids, respectively. A, B and C SNX27 interacts with OTULIN. HeLa cells with SNX27 (A and B) or OTULIN (C) overexpression were immunoprecipitated with GFP, Myc or Flag beads, followed immunoblotting with OTULIN (A and B) or SNX27 (C) antibody to detect the endogenous interaction. Immunoblotting was performed at least twice, and one representative figure was shown. D SNX27 direct interacts with OTULIN. Bacterial recombinantly expressed GST-SNX27 coupled to glutathione agarose were incubated with bacterial lysates expressing His-OTULIN. GST pull-down experiment was performed to study the direct interaction of SNX27 and OTULIN. Ponceau read staining and His antibody were used to detect the interaction after immunoblotting. E The last 6 amino acids of OTULIN interacts with SNX27. Flag tagged OUTLIN or OTULIN-d6 (without last 6 amino acids) coupled to Flag-M2 beads were incubated with bacterial lysates expression GST-SNX27. GST antibody were used to detect the interaction after immunoblotting. F The amino acids of OTULIN that responsible for interaction with SNX27. HEK293T cells were transfected with Myc-SNX27 and Flag-OTULIN mutations. Myc antibody was used to detect the interaction after Flag-M2 beads immunoprecipitation and immunoblotting. G The PDZ domain of SNX27 interacts with OTULIN. GST tagged SNX27 or SNX27-dPDZ coupled to glutathione agarose were incubated with bacterial lysates expressing Flag-OTULIN. Flag antibody were used to detect the interaction after immunoblotting. [file 13578_2021_659_MOESM2_ESM.pdf]

**A**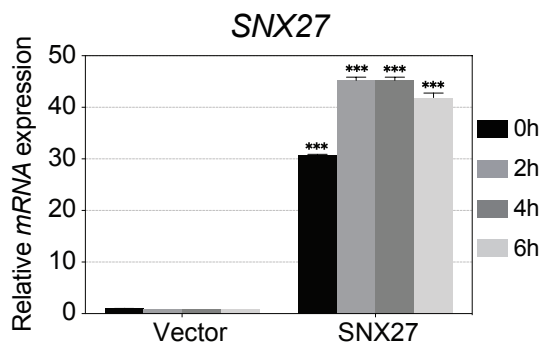**B**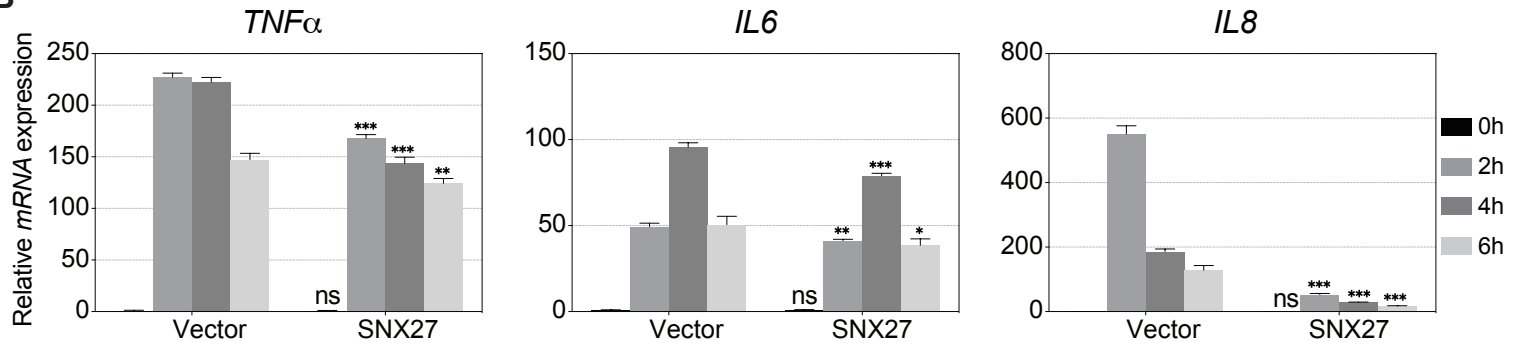**D**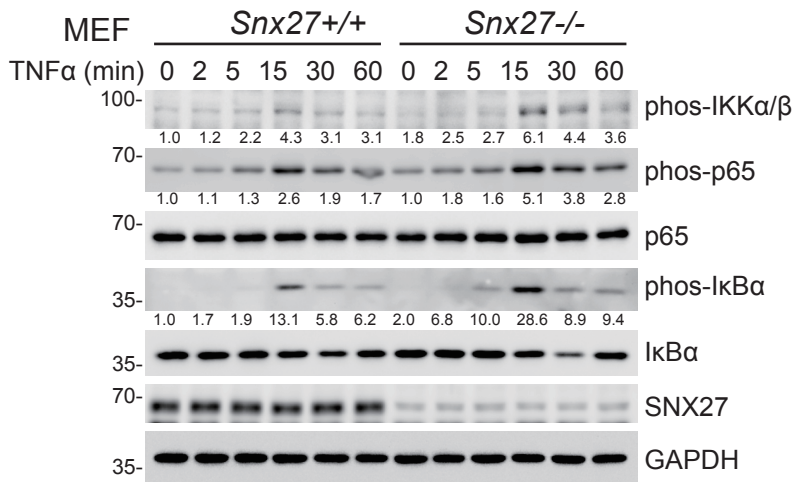**C**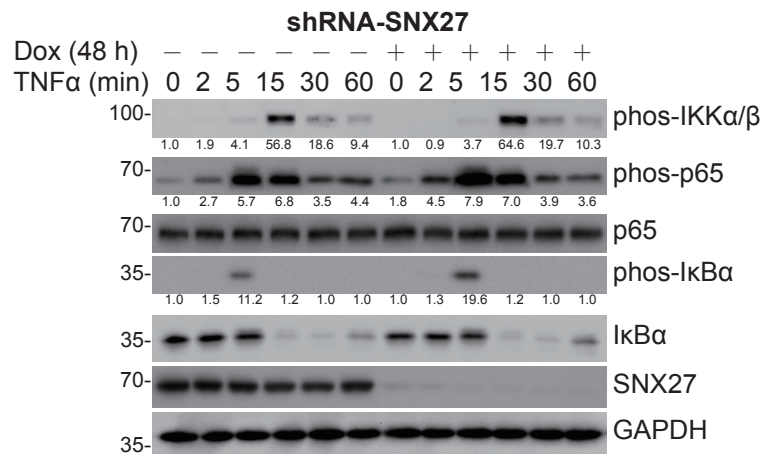

Supplement: Supplementary file 3 — Additional file 3: Figure S3. SNX27 negatively regulates TNFα-induced NF-κB signalling activation. A and B Overexpression of SNX27 inhibits TNFα-induced NF-κB signalling activation. Quantitative real-time PCR was used to check the expression of SNX27 (A) and target genes of TNFα signalling (B). Significant differences compared to control were calculated using multiple t-tests. The graphs showed mean ± SD, n = 3. ns indicates not significant; * indicates p < 0.05, ** indicates p < 0.01; *** indicates p < 0.001. C Knockdown of SNX27 potentiates TNFα-induced NF-κB signalling activation. HeLa cells with doxycycline induced SNX27 knockdown were treated with TNFα at indicated time points. Immunoblotting was performed as Fig. 2A to check the phosphorylation of IκBα, IKKα/β and p65. The relative protein expression level was calculated by ImageJ and labelled below each blot. The value of time 0 of control cells was set as 1. Immunoblotting was performed at least twice, and one representative figure was shown. D Knockout of Snx27 in MEF cells potentiates TNFα-induced NF-κB signalling activation. Immunoblotting was performed and the relative protein expression was calculated as Figure S3C. [file 13578_2021_659_MOESM3_ESM.pdf]

**A**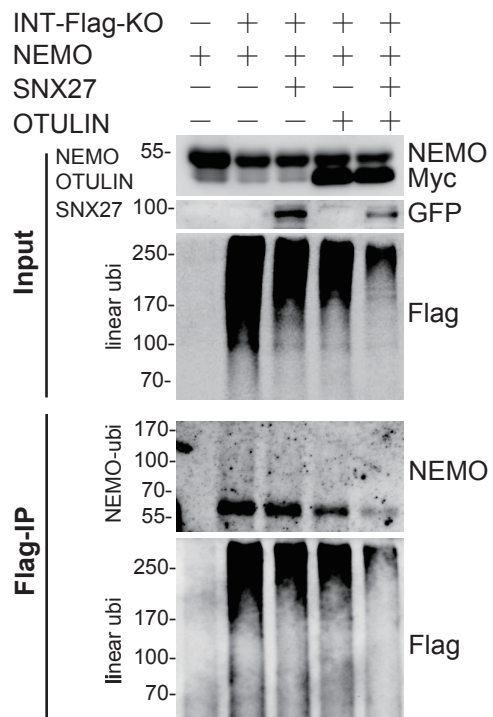**B**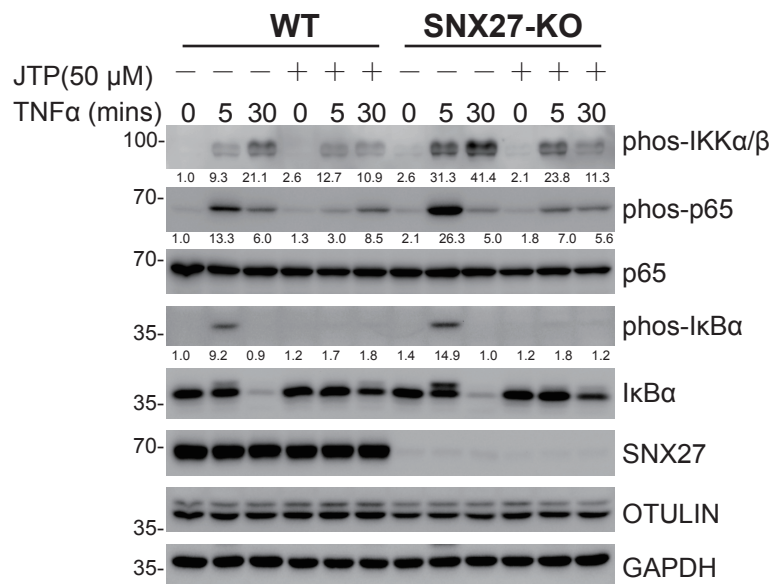

Supplement: Supplementary file 4 — Additional file 4: Figure S4. SNX27 inhibits LUBAC-mediated linear polyubiquitination and NF-κB signalling activation. A SNX27 inhibits linear polyubiquitination of NEMO. HEK293T cells transfected with indicated plasmids were lysed in 1% SDS buffer, followed with 10 × dilution and Flag-resin immunoprecipitation. NEMO antibody was used to detect its linear polyubiquitination. Immunoblotting was performed as Fig. 3A. Immunoblotting was performed at least twice, and one representative figure was shown. B LUBAC activity is required for SNX27-mediated inhibition of TNFα-induced NF-κB signalling activity. HeLa cells with CRISPR/Cas9 mediated SNX27 knockout were treated with JTP-0819958 and/or TNFα for indicated time points. Phosphorylated IκBα, IKKα/β and p65 were used to indicate TNFα-induced NF-κB signalling activity. The value of time 0 of control cells was set as 1. The relative protein expression level was calculated by ImageJ and labelled below each blot. [file 13578_2021_659_MOESM4_ESM.pdf]

**A**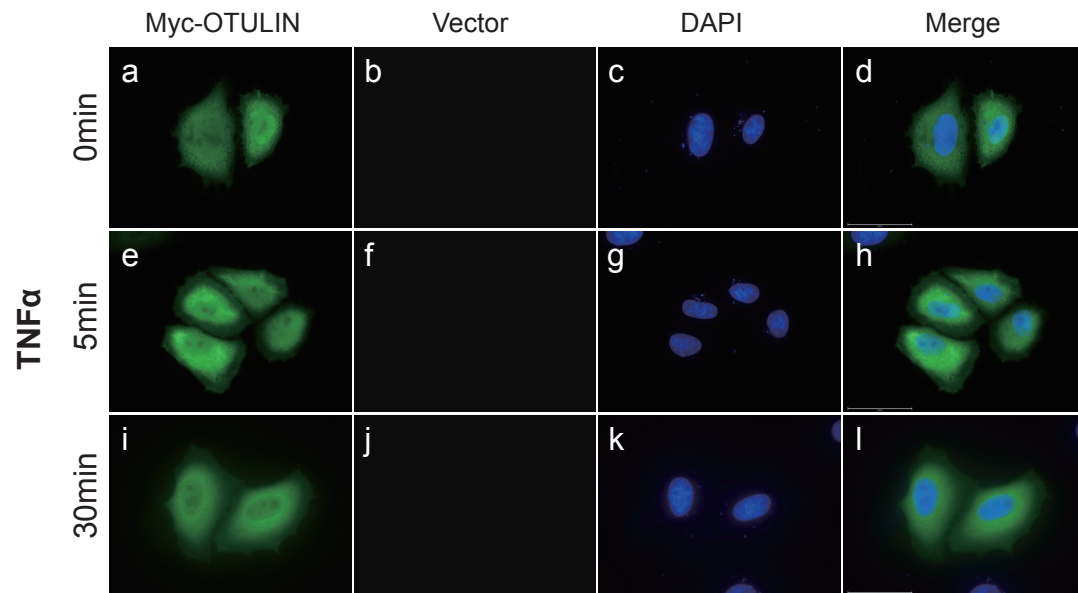**B**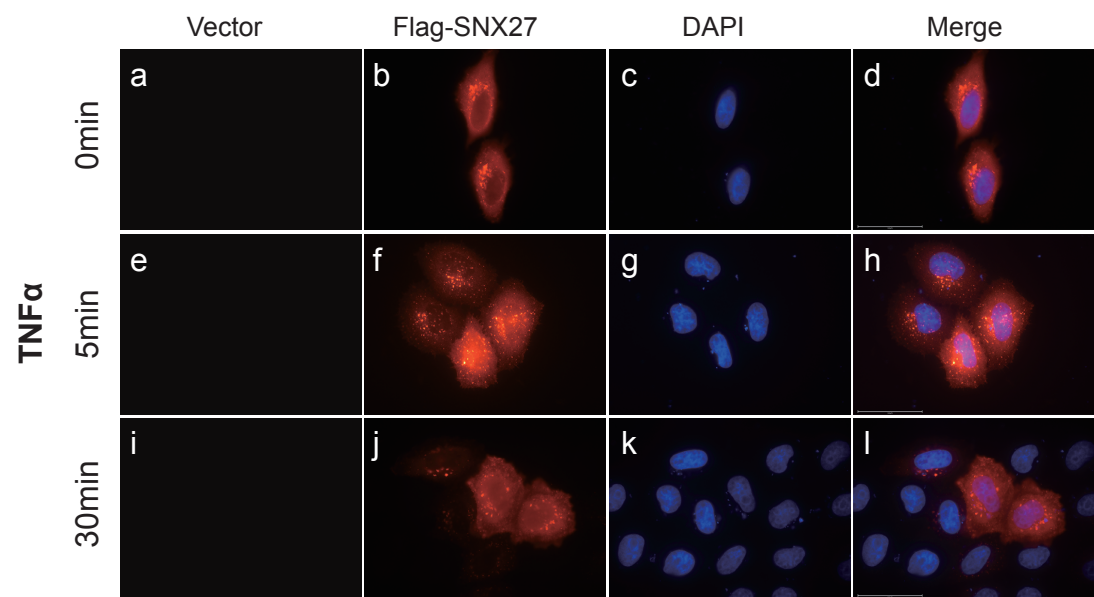**C**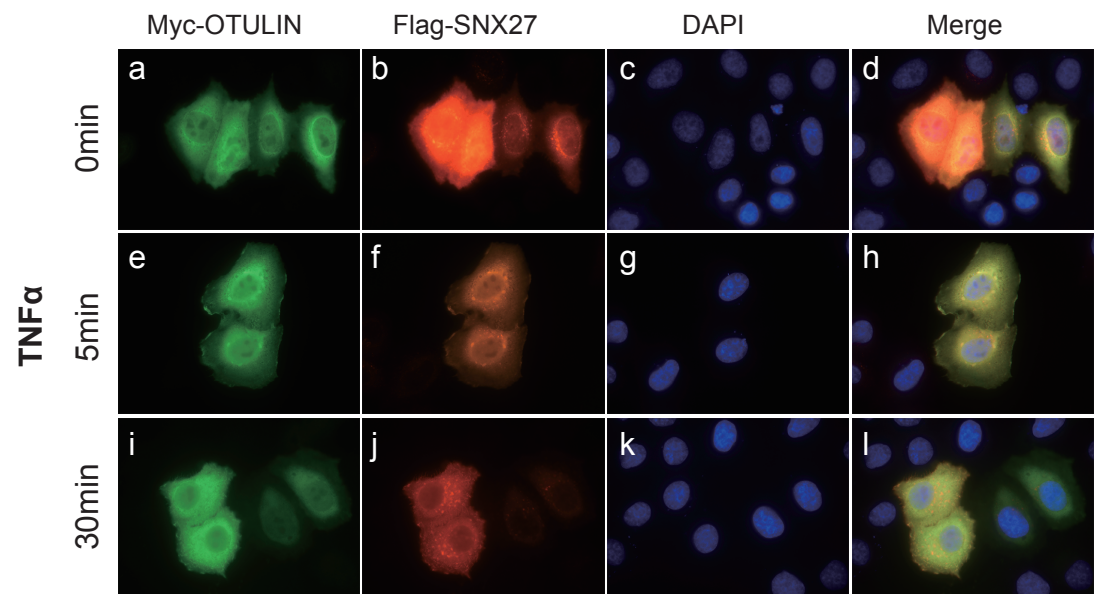**D**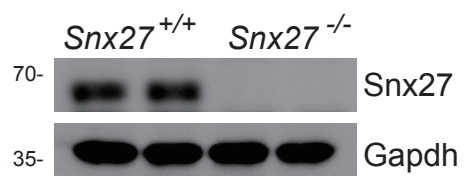

Supplement: Supplementary file 5 — Additional file 5: Figure S5. SNX27 facilitates TNFα-induced membrane localization of OTULIN. A, B and C TNFα-induced membrane localization of OTULIN is potentiated by SNX27 expression. HeLa cells transfected with Myc-OTULIN (A), Flag-SNX27 (B) and Flag-SNX27 and Myc-OTULIN together (C) were treated with TNFα for indicated time points. Cells were fixed and stained with Flag and Myc antibodies. D Validation of SNX27 expression in SNX27 knockout MEF cells. Wild type and SNX27 knockout MEF cells were lysed for immunoblotting. [file 13578_2021_659_MOESM5_ESM.pdf]
